# Jabuticaba (*Plinia* sp.) and Its Phenolic Microencapsulated Peel Extract Attenuate Fat Accumulation and Improve Redox Balance and Lipid Profile in C57BL/6 Mice Fed a High-Fat High-Fructose Diet

**DOI:** 10.3390/ph19081158

**Published:** 2026-07-25

**Authors:** Kelly Aparecida Dias, Livya Alves Oliveira, Stephanie Michelin Santana Pereira, Marcella Duarte Villas Mishima, Lívia Morato Nicoli, Luiz Otávio Guimarães-Ervilha, Camilo José Ramírez-López, Renner Philipe Rodrigues Carvalho, Mariana Machado-Neves, Ceres Mattos Della Lucia

**Affiliations:** 1 Department of Nutrition and Health, Universidade Federal de Viçosa, Av. Peter Henry Rolfs, s/n, Campus Universitário, Viçosa 36570-900, Minas Gerais, Brazil; kelly.dias@ufv.br (K.A.D.); livya.oliveira@ufv.br (L.A.O.); stephanie.pereira@ufv.br (S.M.S.P.); marcella.mishima@ufv.br (M.D.V.M.); livia.nicoli@ufv.br (L.M.N.); 2 Department of General Biology, Universidade Federal de Viçosa, Av. Peter Henry Rolfs, s/n, Campus Universitário, Viçosa 36570-900, Minas Gerais, Brazil; luiz.ervilha@ufv.br (L.O.G.-E.); camilo.lopez@ufv.br (C.J.R.-L.); renner.carvalho@ufv.br (R.P.R.C.); mariana.mneves@ufv.br (M.M.-N.)

**Keywords:** bioactive compounds, anthocyanins, oxidative stress, adipose tissue dysfunction, Brazilian berry

## Abstract

**Background/Objectives**: Obesity induced by high-fat high-fructose (HFHF) diets is associated with adipose tissue expansion, dysregulated adipogenesis, chronic low-grade inflammation, and oxidative stress. Bioactive compounds have emerged as promising nutritional strategies to mitigate these alterations, although their stability and bioavailability remain limiting factors. This study investigated the effects of different forms of jabuticaba (*Plinia* sp.) consumption—whole fruit, freeze-dried peel, and phenolic microencapsulated peel extract—on adipose tissue remodeling, oxidative stress, and metabolic parameters in HFHF-fed mice. **Methods**: Male C57BL/6 mice were fed an HFHF diet for six weeks, followed by six weeks of intervention with the jabuticaba formulations. **Results**: HFHF feeding induced significant metabolic alterations, evidenced by increased body weight gain, abdominal perimeter, Lee index, and fasting glucose levels. Jabuticaba intake, regardless of formulation, reduced abdominal perimeter, final body weight, and Lee index (*p* < 0.05) without affecting food intake. Treatment also improved metabolic biomarkers, reducing serum glucose, LDL cholesterol, and triglycerides, while increasing HDL cholesterol (*p* < 0.05). In addition, jabuticaba decreased visceral and total adipose tissue mass, attenuated adipocyte hypertrophy, and enhanced antioxidant defenses, leading to reduced oxidative damage in adipose tissue. **Conclusions**: All the jabuticaba formulations exerted protective metabolic effects; the whole fruit showed more consistent systemic metabolic improvements, whereas the microencapsulated peel extract demonstrated greater efficacy in modulating adipose tissue.

## 1. Introduction

Obesity, particularly when associated with excessive abdominal fat deposition, has become one of the major current public health challenges [1]. Approximately 40% of the world’s population is overweight or obese, and this number is estimated to exceed 4 billion people by 2035 [2]. Excessive fat deposition in visceral white adipose tissue (vWAT) is the central pathological finding in this condition, indicating metabolic alterations. Excessive consumption of high-fat, high-fructose (HFHF) diets promotes a metabolic overload that triggers the accelerated expansion of white adipose tissue, characterized by adipocyte hypertrophy. The increased size (hypertrophy) of adipocytes increases the secretion of pro-inflammatory cytokines and favors the establishment of a low-grade systemic inflammatory state [3,4].

The initial activation of these processes is directly associated with energy imbalance, resulting in intracellular lipid accumulation and increased generation of reactive oxygen species (ROS) [5]. Obesity is also associated with increased systemic oxidative stress, intensified by Nicotinamide Adenine Dinucleotide Phosphate Hydride (NADPH) oxidases, alterations in oxidative phosphorylation, stimulation of protein kinase C, and hyperleptinemia. In parallel, oxidative stress exacerbates inflammation, compromising lipid metabolism by favoring the accumulation of white adipose tissue and stimulating the proliferation of pre-adipocytes [6].

The adipose tissue dysfunction impacts multiple metabolic systems, intensifying risk factors for the development of insulin resistance, type 2 diabetes, cardiovascular disorders, and non-alcoholic fatty liver disease [7]. Given these impacts, there is growing interest in nutritional strategies that utilize bioactive compounds capable of modulating inflammatory, oxidative stress, as well as reducing their metabolic consequences. Jabuticaba (*Plinia* sp.), a Brazilian berry rich in phenolic compounds and anthocyanins, has been studied for its positive effects on biological variables, including anti-inflammatory, antioxidant, hypoglycemic, and hypolipidemic activities [8,9]. Furthermore, jabuticaba has a high content of dietary fiber (3.2% to 4.8%, based on fresh matter) [10]. Previous studies showed that jabuticaba extract rich in polyphenols (100 mg GAE/kg BW/day) reduced weight gain and adipose tissue weight, as well as decreasing adipocyte hypertrophy and gene expression of pro-inflammatory cytokines in the liver and adipose tissue of mice induced by a high-fat diet [11]. Furthermore, freeze-dried jabuticaba peel and seeds reduced adipocyte size and the expression of pro-inflammatory cytokines in the visceral adipose tissue of mice fed a high-fat diet [12].

Although polyphenols and anthocyanins offer several benefits, they can undergo degradation during food processing and storage, reducing their bioavailability [13]. Microencapsulation emerges as an effective strategy to protect these compounds, increasing their stability, bioavailability, solubility, and applicability in food and nutraceutical products [13,14]. Despite growing evidence of the metabolic effects of jabuticaba, the relative contribution of its distinct dietary forms remains insufficiently understood. In particular, it is still unclear whether these effects are primarily driven by the synergistic interactions of the whole fruit matrix or are mainly attributable to its phenolic-rich fraction. In addition, the extent to which different processing strategies, such as freeze-drying and microencapsulation, modulate the biological effects of jabuticaba-derived compounds in metabolically relevant tissues remains to be elucidated. Therefore, this study investigated the effects of different forms of jabuticaba consumption (whole fruit, peel, and microencapsulated peel extract) on adipose tissue in mice fed an HFHF diet. The comparison among these three presentations allows evaluation of biologically distinct jabuticaba-derived matrices that differ in processing, composition, and dietary incorporation.

## 2. Results

### 2.1. Induction Period

**Figure 1:**
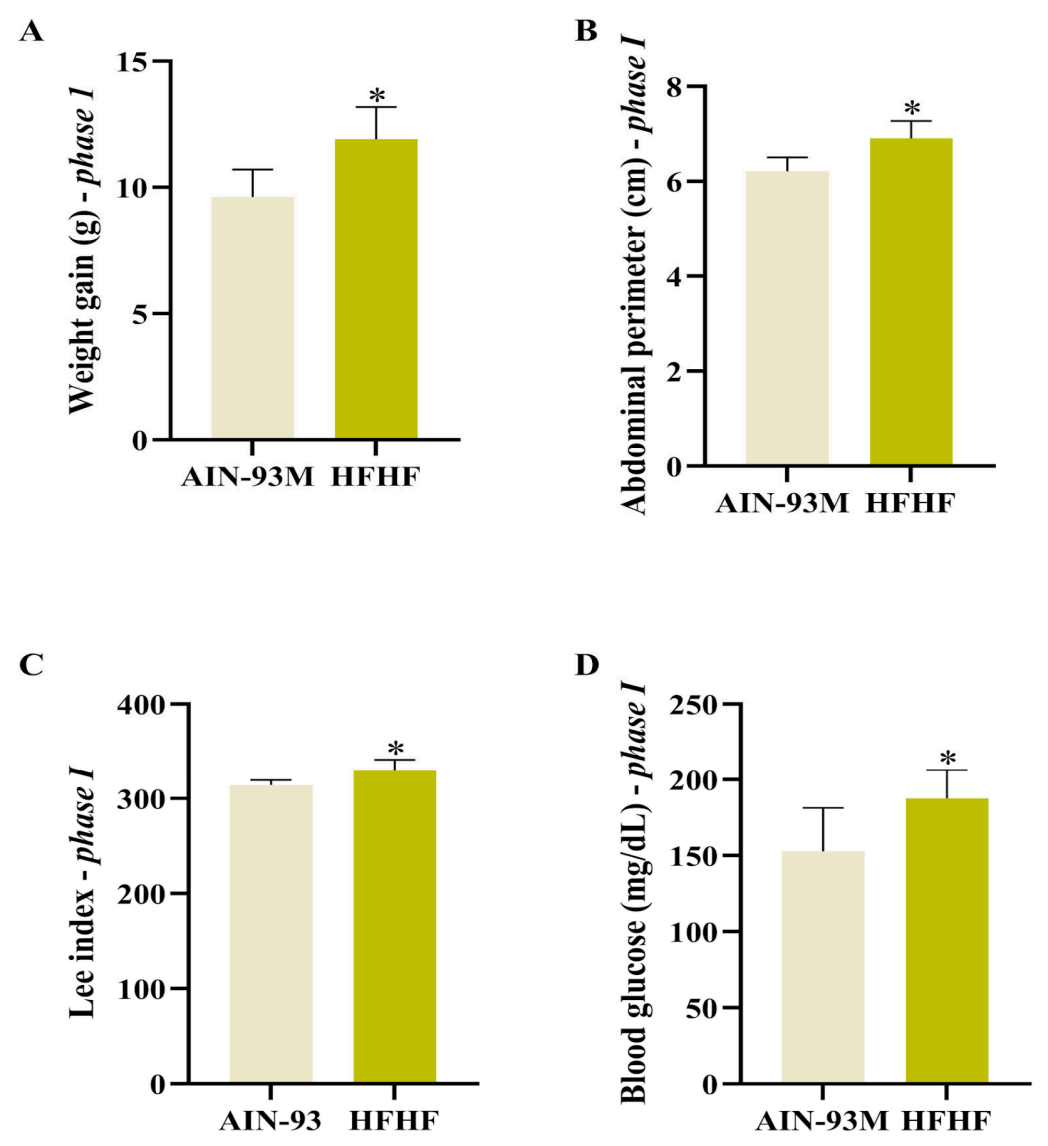
Murinometric measurements of the animals after 6 weeks of the induction period: (**A**) weight gain (g); (**B**) abdominal perimeter (cm); (**C**) Lee index; (**D**) blood glucose (mg/dL). Values are mean ± standard deviation. *p* values < 0.05 are shown as * in the figure according to the unpaired *t* test. AIN-93M: control group; HFHF: diet rich in saturated fatty acids and fructose.

After six weeks of the induction period, animals fed the high-fat, high-fructose (HFHF) diet showed a significant increase in all the parameters evaluated compared to the AIN-93M group (Figure 1). Body weight gain (Figure 1A) was significantly greater in the HFHF group. Similarly, abdominal circumference increased (Figure 1B), suggesting greater central adiposity. The Lee index (Figure 1C), considered an indicator of obesity, was also significantly elevated in animals fed the HFHF diet, reinforcing the development of an obese phenotype. Furthermore, fasting blood glucose levels (Figure 1D) were significantly higher in the HFHF group (*p* < 0.05) (Figure 1).

### 2.2. Treatment Period

#### 2.2.1. Identification of Phenolic Compounds

**Figure 2:**
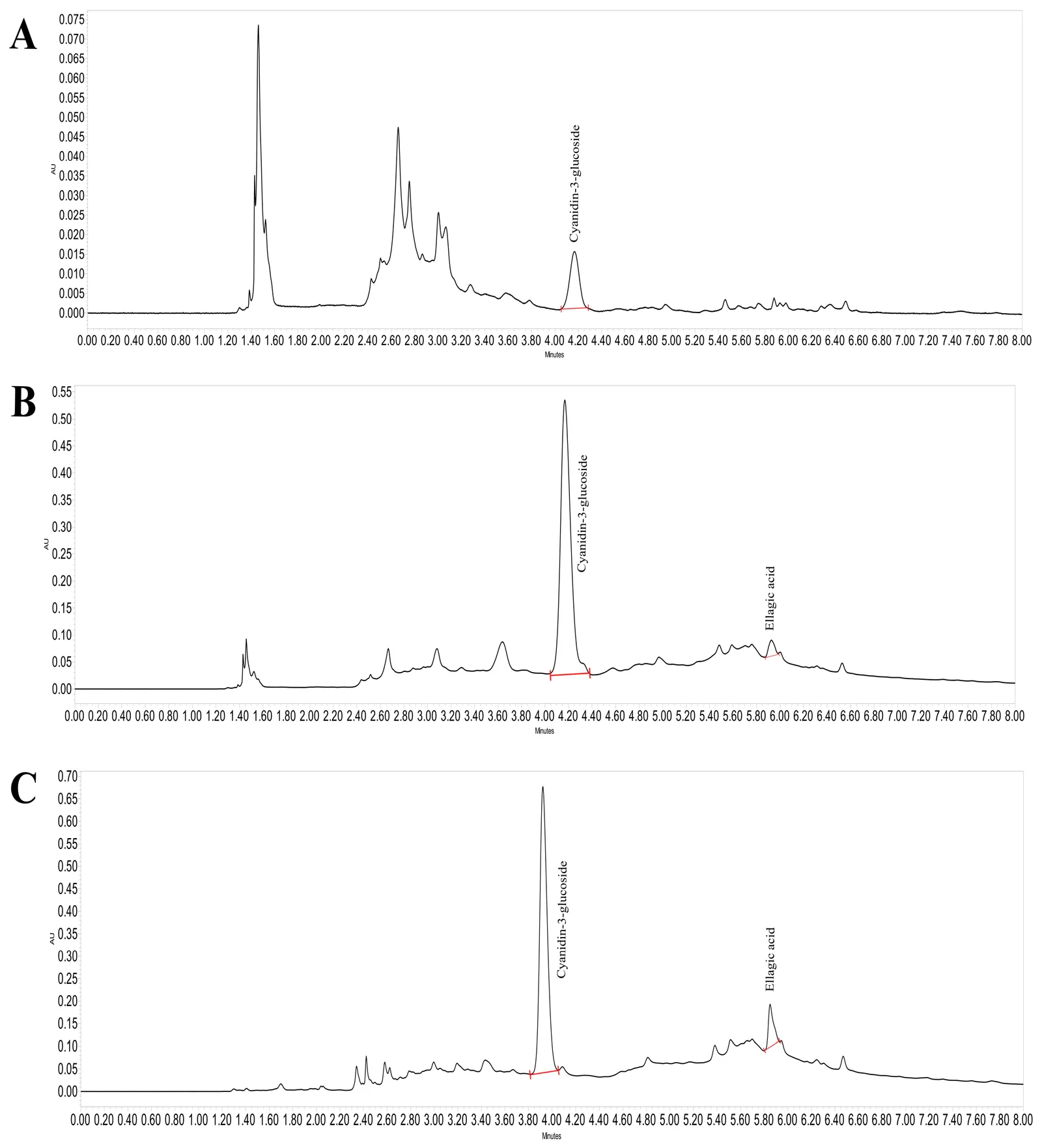
Identification of the main phenolic compounds in jabuticaba by UPLC/PDA analysis: (**A**) whole fruit; (**B**) jabuticaba peel; (**C**) jabuticaba peel extract.

Cyanidin-3-glucoside was identified in all the samples analyzed, including the whole fruit, the peel, and the liquid extract of the peel. Ellagic acid was identified in both peel-derived samples, both in the peel and in the liquid extract of the peel, but was not detected in the whole fruit. Representative chromatograms of the analyzed samples are presented in Figure 2.

#### 2.2.2. Effect of Jabuticaba on Murinometric Measurements, Food Consumption, and Biochemical Variables

**Figure 3:**
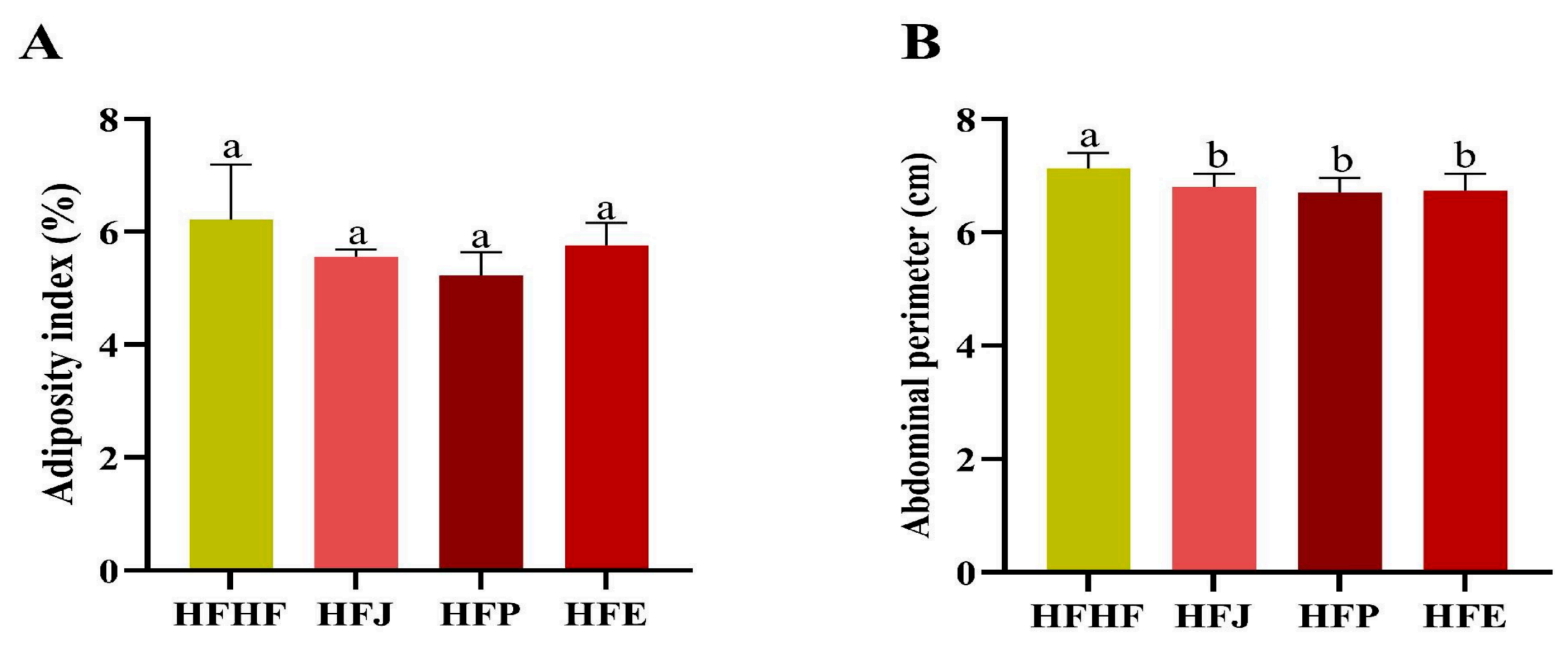
Effect of jabuticaba (whole fruit, peel and microencapsulated peel extract) on the (**A**) abdominal perimeter and (**B**) adiposity index after 6 weeks of induction phase and after 6 weeks receiving the experimental diets. Values are expressed as means ± standard deviation (SD). *n* = 9/group. a,b Treatment group means not indicated by the same letter are significantly different (*p* < 0.05) according to the Newman–Keuls test. HFHF: diet rich in saturated fatty acids and fructose; HFJ: diet rich in saturated fatty acids and fructose + freeze-dried whole jabuticaba fruit; HFP: diet rich in saturated fatty acids and fructose + freeze-dried jabuticaba peel; HFE: diet rich in saturated fatty acids and fructose + microencapsulated peel extract.

Jabuticaba supplementation did not alter the adiposity index compared with the HFHF group (*p* > 0.05) (Figure 3A). In contrast, all three forms of jabuticaba (whole fruit, peel, and microencapsulated peel extract) reduced the abdominal perimeter relative to the HFHF group (*p* < 0.05) (Figure 3B).

**Figure 4:**
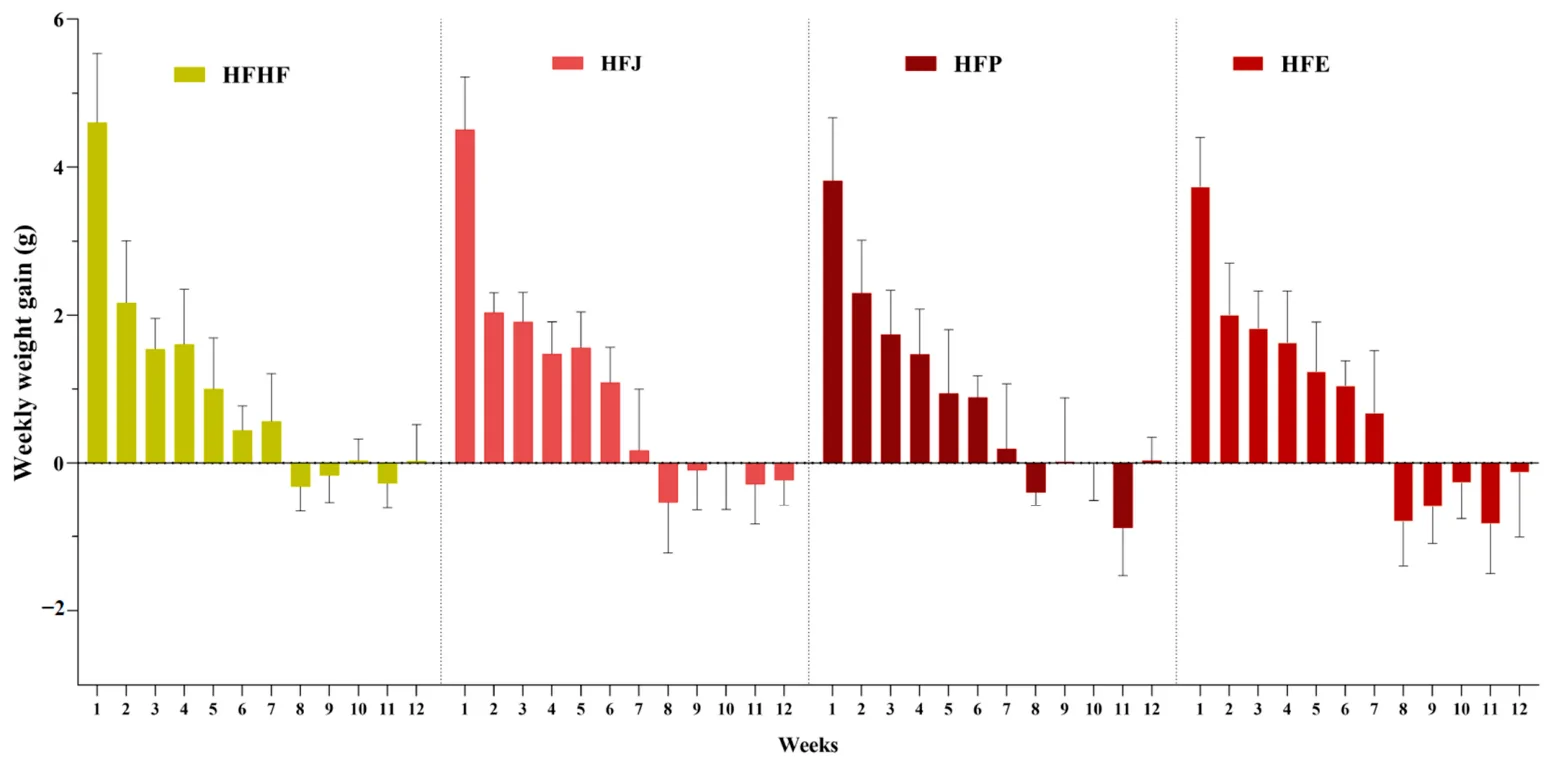
Effect of jabuticaba (whole fruit, peel and microencapsulated peel extract) on weekly body weight gain. HFHF: diet rich in saturated fatty acids and fructose; HFJ: diet rich in saturated fatty acids and fructose + freeze-dried whole jabuticaba fruit; HFP: diet rich in saturated fatty acids and fructose + freeze-dried jabuticaba peel; HFE: diet rich in saturated fatty acids and fructose + microencapsulated peel extract.

Regarding murinometric variables, the initial body weight did not differ among the experimental groups (*p* > 0.05). However, the intake of jabuticaba in all three forms evaluated (whole fruit, peel, and microencapsulated peel extract) resulted in a lower final body weight compared with the HFHF group (*p* < 0.05). Nevertheless, body weight gain did not differ among the groups (Figure 4).

Similarly, the Lee index was reduced in the jabuticaba-treated groups relative to the HFHF group (*p* < 0.05). Food intake and energy intake did not differ among the groups (*p* > 0.05), indicating that the reductions in body weight and Lee index were not attributable to decreased energy consumption (Table 1).

Concerning biochemical parameters, the intake of jabuticaba in all three forms reduced serum glucose levels compared with the HFHF group (*p* < 0.05). Total cholesterol levels did not differ among the experimental groups (*p* > 0.05). In contrast, LDL cholesterol was lower, and HDL cholesterol was higher in the jabuticaba groups relative to the HFHF group (*p* < 0.05). Serum triglyceride levels were also reduced following jabuticaba supplementation in all three forms evaluated (*p* < 0.05). Among the treated groups, mice receiving whole jabuticaba exhibited the lowest triglyceride concentration (67.74 ± 1.38), differing from the peel-supplemented group (72.60 ± 3.82), whereas the group treated with the microencapsulated peel extract (HFE) (70.07 ± 2.74) did not differ from either the whole fruit or peel groups (*p* > 0.05) (Table 1).

#### 2.2.3. Effect of Jabuticaba on Adipose Tissue Weights

**Figure 5:**
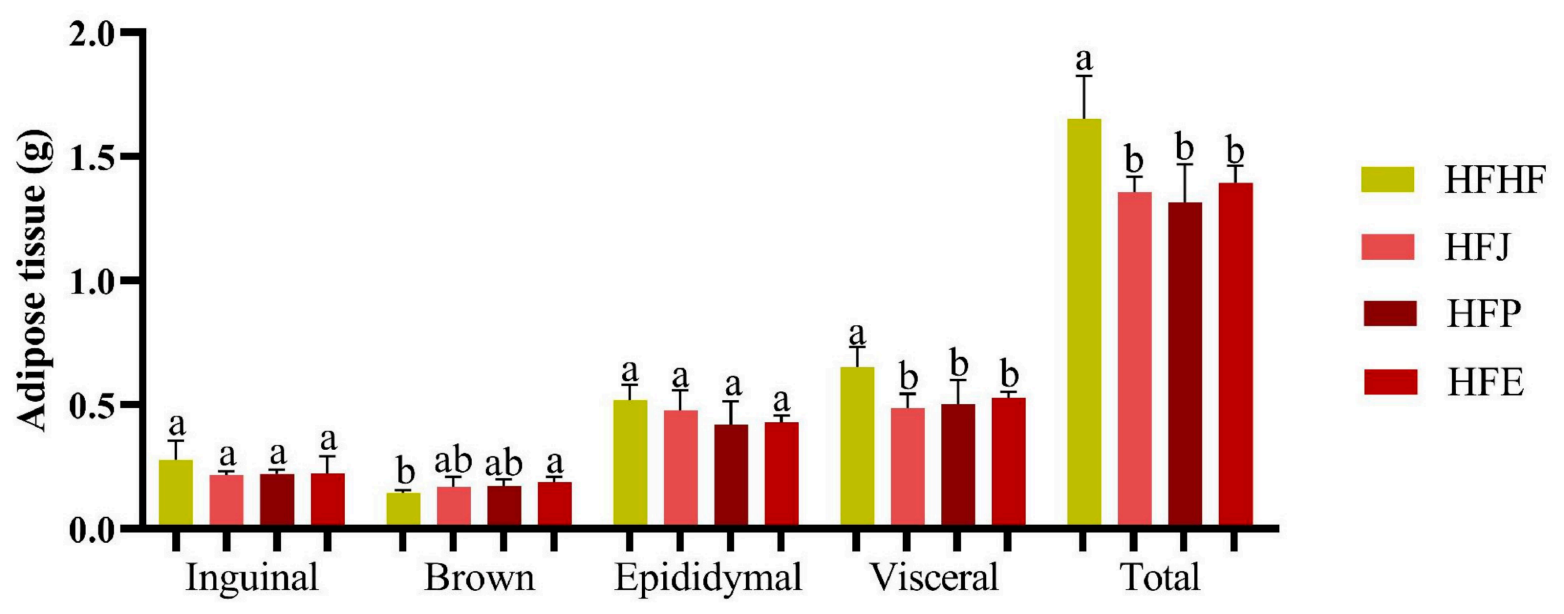
Distribution of different adipose tissue compartments in mice after 6 weeks of HFHF induction followed by 6 weeks of treatment with jabuticaba in different forms. HFHF: diet rich in saturated fatty acids and fructose; HFJ: diet rich in saturated fatty acids and fructose + freeze-dried whole jabuticaba fruit; HFP: diet rich in saturated fatty acids and fructose + freeze-dried jabuticaba peel; HFE: diet rich in saturated fatty acids and fructose + microencapsulated peel extract. Values are expressed as means ± standard deviation (SD). *n* = 9/group. a,b Treatment group means not indicated by the same letter are significantly different (*p* < 0.05) according to the Newman–Keuls test.

The intake of jabuticaba in all three forms evaluated (whole fruit, peel, and microencapsulated peel extract) reduced the visceral and total adipose tissue (*p* < 0.05). The brown adipose tissue was increased in the HFE group, compared to the HFHF group (*p* < 0.05), and there was no difference among the groups for inguinal and epididymal adipose tissues (*p* > 0.05) (Figure 5).

#### 2.2.4. Effects of Jabuticaba on Glucose Tolerance

**Figure 6:**
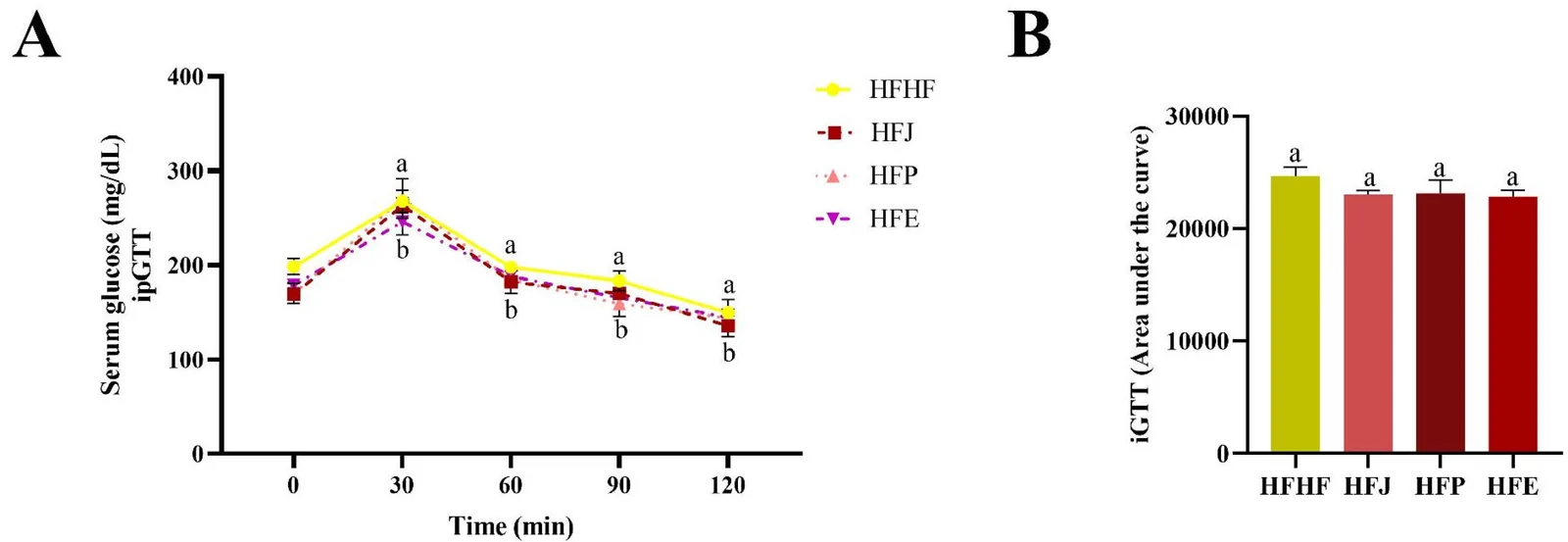
Glucose tolerance of C57BL/6 mice after 6 weeks of the induction phase and 6 weeks of receiving the experimental diets with freeze-dried whole fruit, freeze-dried jabuticaba peel, or peel extract microencapsulated: (**A**) glucose tolerance test. Values are expressed as means ± standard deviation (SD); (**B**) area under the curve of the glucose tolerance test. a,b Treatment group means not indicated by the same letter are significantly different (*p* < 0.05) according to the Newman–Keuls test. HFHF: diet rich in saturated fatty acids and fructose; HFJ: diet rich in saturated fatty acids and fructose + freeze-dried whole jabuticaba fruit; HFP: diet rich in saturated fatty acids and fructose + freeze-dried jabuticaba peel; HFE: diet rich in saturated fatty acids and fructose + microencapsulated peel extract.

Two-way ANOVA showed significant effects of time and group on serum glucose levels during the ipGTT, with the HFHF group exhibiting higher glucose levels compared with the treatment groups (Figure 6A). However, no significant differences among groups were observed in overall glycemic exposure, as assessed by the area under the curve (AUC) (Figure 6B).

#### 2.2.5. Effect of Jabuticaba on Oxidative Stress Markers and Antioxidant Enzyme Activities

**Figure 7:**
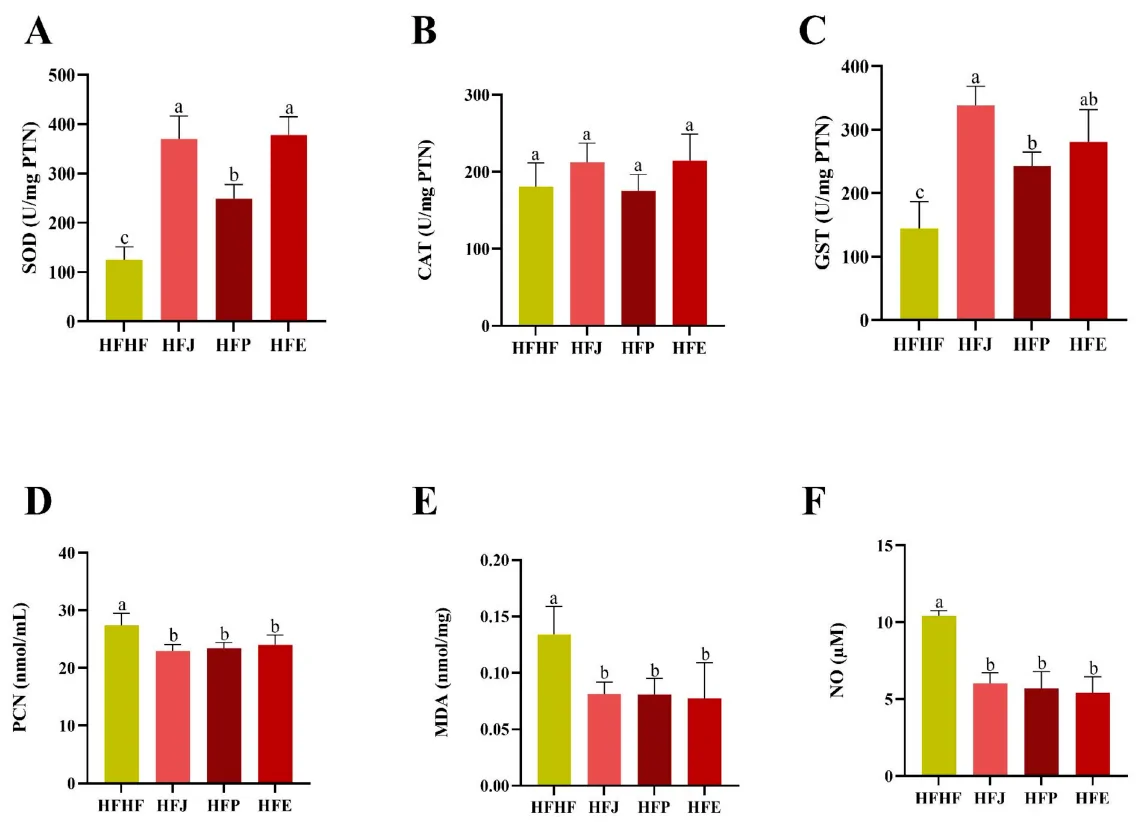
Oxidative stress in adipose tissue homogenate of mice after 6 weeks of induction phase and after 6 weeks receiving the experimental diets with freeze-dried whole fruit, freeze-dried jabuticaba peel or peel extract microencapsulated: (**A**) superoxide dismutase; (**B**) catalase; (**C**) glutathione-S-transferase; (**D**) carbonyl protein; (**E**) malondialdehyde and (**F**) nitric oxide. Values are expressed as means ± standard deviation (SD). *n* = 9/group. a–c Treatment group means not indicated by the same letter are significantly different (*p* < 0.05) according to the Newman–Keuls test. HFHF: rich in saturated fatty acids and fructose; HFJ: diet rich in saturated fatty acids and fructose + freeze-dried whole jabuticaba fruit; HFP: diet rich in saturated fatty acids and fructose + freeze-dried jabuticaba peel; HFE: diet rich in saturated fatty acids and fructose + microencapsulated peel extract; SOD: superoxide dismutase; CAT: catalase; GST: glutathione-S-transferase; PCN: Carbonyl protein; MDA: Malondialdehyde; NO: Nitric oxide.

The intake of jabuticaba in all three forms evaluated (whole fruit, peel, and microencapsulated peel extract) increased SOD and GST activities compared with the HFHF group, and also reduced carbonylated proteins, malondialdehyde, and nitric oxide levels (*p* < 0.05). Among the groups that received jabuticaba, the group supplemented with the freeze-dried whole jabuticaba fruit (HFJ) showed higher SOD and GST activities than the group fed the peel (HFP) (*p* < 0.05) (Figure 7).

#### 2.2.6. Effect of Jabuticaba on Adipose Tissue Histomorphometry

**Figure 8:**
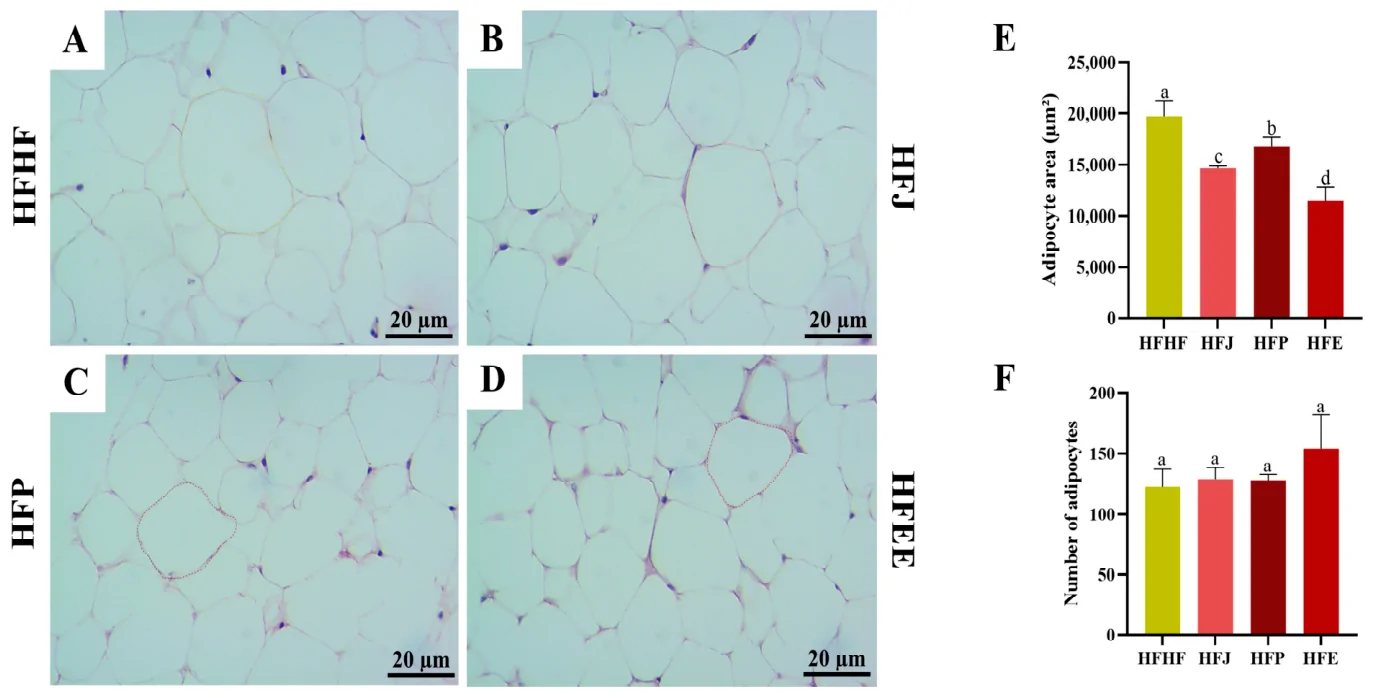
Adipose tissue photomicrographs of the experimental groups. (**A**) HFHF: high-fat saturated-fructose diet; (**B**) HFJ: high-fat saturated-fructose diet + freeze-dried whole jabuticaba fruit; (**C**) HFP: high-fat saturated-fructose diet + freeze-dried jabuticaba peel; (**D**) HFE: high-fat saturated-fructose diet + microencapsulated peel extract; (**E**) adipocyte area; (**F**) and number of adipocytes. The dashed line indicates the perimeter used to delimit the adipocyte area for morphometric analysis. Values are expressed as means ± standard deviation (SD). *n* = 9/group. a–d Treatment group means not indicated by the same letter are significantly different (*p* < 0.05) according to the Newman–Keuls test.

The intake of jabuticaba in all three forms evaluated (whole fruit, peel, and microencapsulated peel extract) significantly reduced adipocyte area compared with the HFHF group (*p* < 0.05). Among the jabuticaba-treated groups, supplementation with the microencapsulated peel extract (HFE) resulted in the lowest adipocyte area, followed by HFJ and HFP (Figure 8E). The number of adipocytes (Figure 8F) did not differ between the jabuticaba-treated groups and the HFHF group.

#### 2.2.7. Pearson Correlation Analysis

**Figure 9:**
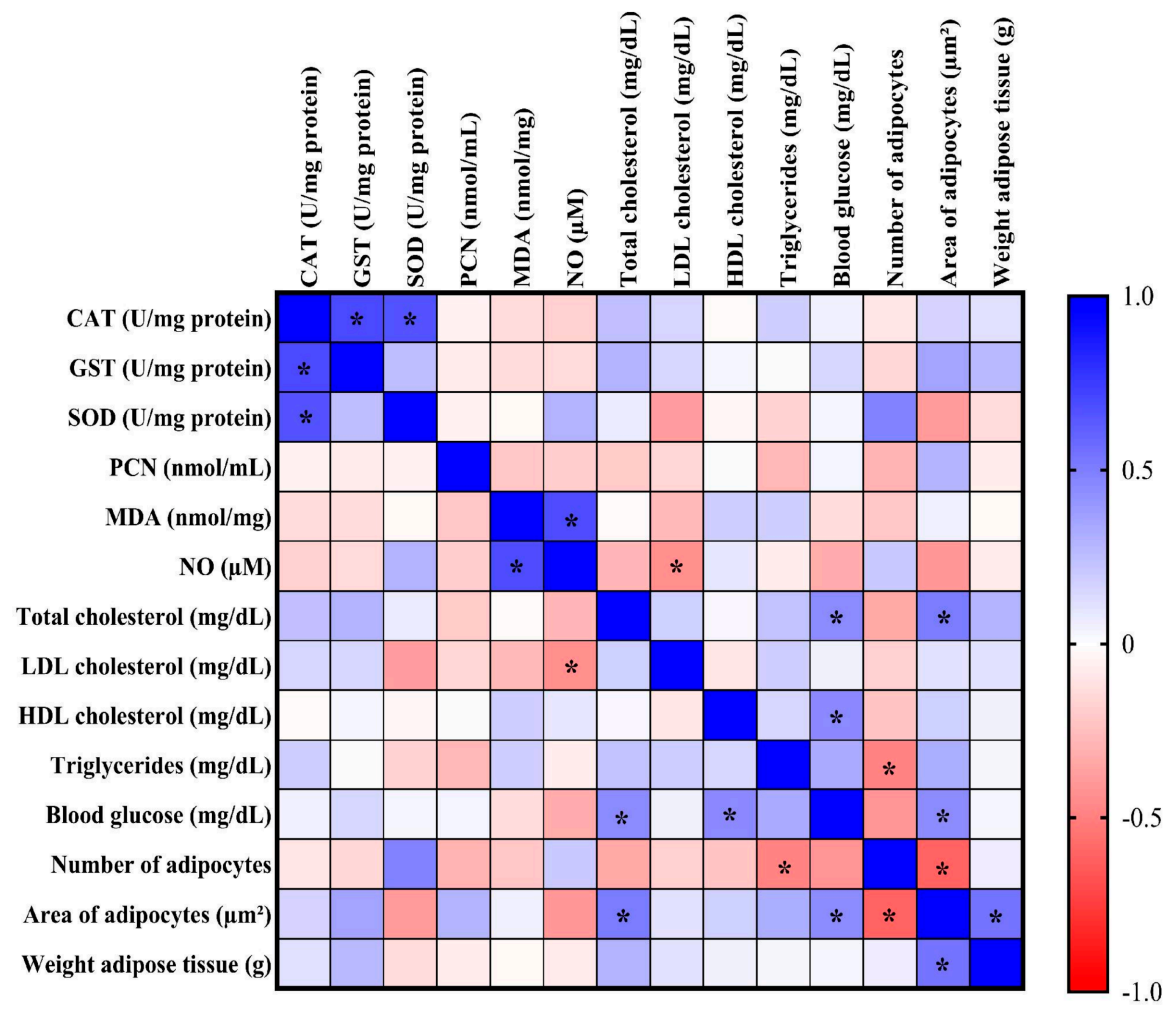
Heatmap of Pearson correlation analysis. CAT, catalase; GST, glutathione-S-transferase; SOD, superoxide dismutase; PCN, carbonyl protein; MDA, malondialdehyde; NO, nitric oxide; LDL, low-density lipoprotein; HDL, high-density lipoprotein. * Means statistically significant difference (*p* < 0.05).

Pearson correlation analysis was used to assess associations among oxidative stress biomarkers, biochemical variables, histological parameters, and body weight, in order to explore potential relationships within the dataset. Positive correlations were observed between catalase and glutathione (r = 0.696), catalase and superoxide dismutase (r = 0.659), malondialdehyde and nitric oxide (r = 0.683), total cholesterol and blood glucose (r = 0.448), total cholesterol and adipocyte area (r = 0.509), HDL cholesterol and blood glucose (r = 0.452), blood glucose and adipocyte area (r = 0.443), adipocyte area and adipose tissue weight (r = 0.539). Negative correlations were found between nitric oxide and LDL cholesterol (r = −0.439), triglycerides and number of adipocytes (r = −0.482), and number of adipocytes and adipocyte area (r = −0.604) (Figure 9).

## 3. Discussion

Jabuticaba (*Plinia* sp.), a Brazilian berry, represents a source of phytochemicals with described nutritional benefits, accumulating considerable levels of phenolic compounds such as anthocyanins, mainly cyanidin-3-glucoside, responsible for its dark color [9]. Furthermore, it has a high content of dietary fiber, which is also associated with its beneficial potential. In the present study, the consumption of jabuticaba at a dosage of 200 mg of polyphenols per 100 g of diet, in all the forms evaluated (whole fruit, peel, or microencapsulated peel extract), was able to attenuate the metabolic dysfunction induced by the high-fat-fructose diet (HFHF), promoting reductions in total and visceral adipose tissue, decrease adipocyte area, reduction in oxidative stress, and increased antioxidant activity in C57BL/6 mice.

The dietary dose of 200 mg of total phenolics per 100 g of diet (equivalent to approximately 280 mg/kg/day in mice) corresponds to an estimated 22.7 mg/kg/day in humans (approximately 1.6 g/day for a 60 kg adult), based on body surface area (BSA) conversion according to the method described by Nair and Jacob [15], applying correction factor (Km) of 3 for mice and 37 for humans. This represents a theoretical interspecies equivalence for comparative purposes.

Jabuticaba-derived products may also differ not only in their phenolic markers, but also in their structural characteristics as dietary matrices. Beyond the marker compounds identified by UPLC-PDA, the three tested matrices differed in composition and processing: the whole fruit included pulp-associated components, the peel represented a phenolic-enriched solid fraction, and the microencapsulated extract consisted of a peel-derived phenolic extract carried in maltodextrin. Although all the treatments were standardized according to total phenolic content, equivalent phenolic doses do not necessarily imply biological equivalence among these formulations. Differences in the overall food matrix, including fiber, carbohydrates, and other non-phenolic constituents, may also have influenced the observed biological responses through complementary or synergistic mechanisms. Therefore, the biological comparisons in this study should be interpreted at the formulation level, rather than being exclusively attributed to phenolic compounds. The physicochemical, technological, and phytochemical characterization of the phenolic microencapsulated peel extract used in this study has been previously reported by our research group [16], providing detailed information on the properties of this formulation.

High-fat diet (HFD) models are widely used to reproduce classical features of obesity and its metabolic complications. Among them, the HFHF diet has been extensively applied due to its ability to induce a consistent metabolic phenotype. Although hepatic alterations are well documented in this model, we sought to determine whether HFHF feeding would also promote consistent alterations in adipose tissue, given its role in systemic metabolic homeostasis. Previous studies have similarly employed this model to evaluate adipose tissue adaptations, including the work by Gómez-García et al. [17], which examined changes in brown adipose tissue in rats fed a high-fat high-fructose diet, and Shum et al. [18], which evaluated the role of the angiotensin II receptor in adipocyte differentiation and adipocyte size regulation in a rat model of insulin resistance induced by a high-fat/high-fructose diet. The six-week HFHF diet induction period successfully reproduced the classic characteristics of diet-induced obesity, including increased body weight, abdominal circumference, Lee index, and hyperglycemia, reinforcing the suitability of this model to investigate nutritional strategies targeting adiposity and metabolic impairment, as consistently reported in recent studies that used combined high-fat, high-fructose diets [19,20,21].

During the treatment phase, jabuticaba supplementation demonstrated a lower final body weight without changes in food intake, suggesting that this effect was independent of reduced caloric consumption. These findings corroborate growing evidence that foods rich in anthocyanins improve energy metabolism, lipid homeostasis, and adipose tissue function independently of caloric intake [22,23,24]. The reduction in the Lee index and abdominal circumference further reinforces the beneficial effect on adiposity distribution, particularly on visceral fat accumulation, which is a determinant of cardiometabolic risk [25,26].

Although the adiposity index showed only a trend towards reduction without statistical significance, the decrease in abdominal circumference observed in the jabuticaba-treated groups indicates a reduction in central adiposity. In addition, total body fat was reduced in all the groups that consumed jabuticaba. Together, these findings suggest that jabuticaba intake may contribute to a decrease in overall fat accumulation, with a more pronounced effect on abdominal fat deposition. Similar results have already been described in the literature, showing that specific anthropometric measures may be more sensitive than global indexes in detecting changes in adiposity associated with dietary interventions [27,28].

The observed changes in biochemical parameters indicate potential systemic metabolic effects associated with jabuticaba consumption. A reduction in fasting glucose levels was observed in all jabuticaba-treated groups. However, this effect was not accompanied by significant differences in overall glucose tolerance, as assessed by the area under the curve (AUC) during the iGTT. Although some treatment groups exhibited lower glucose levels than the HFHF group at specific time points, these differences were not reflected in the integrated glycemic response. Therefore, the results should be interpreted as indicative of an improvement in fasting glycemic levels, without evidence of a significant effect on glucose tolerance.

Although total cholesterol remained unchanged, the reduction in LDL cholesterol and the increase in HDL cholesterol highlight a favorable modulation of lipid metabolism. Obesity is closely associated with hypercholesterolemia, a well-recognized risk factor for the development of cardiovascular diseases (CVD), particularly with respect to LDL-C, which is the main carrier of cholesterol in the circulation. Similarly, elevated triglyceride concentrations also represent an important risk factor for CVD [5]. In the present study, jabuticaba consumption resulted in significant reductions in serum LDL-C levels in mice fed an HFHF diet, corresponding to decreases of 19.9% in the group receiving the whole fruit and 22.4% in the groups treated with the peel and the microencapsulated peel extract. These effects are consistent with previously reported biological activities of polyphenols related to lipid metabolism [29,30].

In addition, triglyceride concentrations were reduced by 18.9%, 11.8%, and 15.3% in the groups supplemented with the whole fruit, peel, and microencapsulated extract, respectively. This reduction in triglyceride levels was negatively correlated with adipocyte number, indicating that lower circulating triglycerides were associated with higher adipocyte cellularity.

The reduction in visceral and total adipose tissue weight observed in mice that received jabuticaba in the three forms evaluated indicates a beneficial effect on adipose tissue accumulation even under a high-fat, high-fructose (HFHF) diet. Visceral adipose tissue is particularly relevant from a metabolic point of view, since its expansion is associated with insulin resistance, dyslipidemia, and low-grade chronic inflammation. Therefore, reductions in this depot are commonly associated with improved metabolic profiles [31,32]. The absence of differences in the weight of inguinal and epididymal adipose tissues suggests that the effects of jabuticaba were specific to each depot. This selective response has already been reported in previous studies that evaluated interventions with polyphenols and may reflect differences in adipocyte metabolism, vascularization, and sensitivity to bioactive compounds in the diet among different adipose tissue depots [33,34]. Notably, the increase in brown adipose tissue mass observed only in the group that received the microencapsulated extract suggests a potential modulation of energy-spending tissues, which may contribute to improved metabolic outcomes without changes in dietary intake.

The reduction in visceral adipose tissue was accompanied by significant improvements in oxidative stress markers and antioxidant defense systems. Diets high in fat and fructose are known to promote the excessive production of reactive oxygen species through mitochondrial dysfunction, NADPH oxidase activation, and chronic inflammation, leading to lipid and protein oxidation [35,36]. In our experimental design, systemic metabolic alterations were evaluated using serum biochemical parameters, including glucose and lipid profile. Thus, the assessment of oxidative stress markers in adipose tissue was complementary to these systemic analyses to better characterize the local mechanisms associated with adipose tissue remodeling. The ingestion of jabuticaba, in the three forms evaluated, increased SOD and GST activities, while reducing levels of malondialdehyde, protein carbonyls, and nitric oxide, suggesting an improvement in redox-related parameters in adipose tissue. These findings are consistent with previous reports describing antioxidant effects of anthocyanin-rich matrices. However, in the present study, the observed improvement in adipose tissue redox-related parameters was inferred from enzymatic antioxidant activities and oxidative damage markers and does not demonstrate modulation of specific redox signaling pathways [37,38].

The whole fruit promoted greater antioxidant enzyme activity than the peel alone, suggesting that non-phenolic components, such as vitamins, minerals, and fiber, may potentiate the antioxidant response [39,40]. Conversely, the microencapsulated extract was the only formulation associated with an increase in brown adipose tissue mass, suggesting that different jabuticaba formulations may exert distinct biological effects depending on the evaluated outcome. However, since phenolic bioavailability was not directly assessed, any explanation based on enhanced absorption or stability should be considered hypothesis-generating rather than demonstrated [14,41,42].

Considering that diets with high energy density favor a positive energy balance, resulting in the increase in cell volume due to excessive lipid storage, jabuticaba intake was able to attenuate the progression of adipocyte hypertrophy induced by a high-fat, high-fructose diet, reducing the area of adipocytes. Under chronic positive energy balance, adipocyte size expands until reaching a critical threshold, after which the recruitment of precursor cells and increases in adipocyte number are triggered [43]. In this study, the number of adipocytes did not differ between the jabuticaba-treated groups and the HFHF group, suggesting that the observed effects were primarily associated with reduced adipocyte hypertrophy. These morphological changes are particularly relevant, since adipocyte hypertrophy is strongly associated with hypoxia, macrophage infiltration, oxidative stress, inflammation, and insulin resistance [44].

Accordingly, exploratory Pearson correlation analysis revealed a negative association between adipocyte area and adipocyte number, as well as positive correlations between adipocyte area and blood glucose levels, total cholesterol, and adipose tissue weight, supporting the integrated interpretation of the relationships among adipocyte morphology and metabolic parameters observed in this study. The more pronounced reduction in adipocyte area observed in the group treated with the extract (HFE) may be related to differences in formulation, including potential effects on phenolic compounds’ stability and bioavailability, although these parameters were not directly assessed. Nevertheless, this finding should be interpreted together with the observation that the whole fruit exhibited greater antioxidant enzyme activity, indicating that each formulation may preferentially influence different physiological responses. Polyphenols and anthocyanins have been shown to suppress lipid accumulation by modulating pathways involved in adipocyte differentiation and lipid storage [45,46].

The present study shows associations with improved adipose tissue metabolic, redox-related, and histomorphometric parameters; however, the inclusion of molecular analyses would further strengthen the understanding of the adipogenic and inflammatory signaling pathways underlying these effects. The absence of molecular analyses prevents direct assessment of signaling pathways such as Nrf2-, NF-κB-, or AMPK-related responses. Furthermore, the lack of hepatic endpoints, adipose lipid metabolism markers, and insulin tolerance testing limits broader metabolic inference. In the present study, it was not possible to perform immunohistochemical assessment of adipogenic markers. Quantitative real-time PCR (RT-qPCR) and additional immunohistochemical approaches should be explored in future investigations. The absence of a control group containing only maltodextrin may also be considered a limitation of the present study. Although maltodextrin is widely used as an inert carrier in spray-dried formulations and is not expected to exhibit antioxidant or anti-inflammatory activity at the concentration used, the absence of a maltodextrin-only control group precludes completely ruling out a potential contribution of the carrier material to the observed effects. Future studies should include a maltodextrin-only control group to definitively address this question. Additionally, the absence of formal botanical authentication of the plant material limits the attribution of the observed biological effects to a taxonomically confirmed *Plinia* species. In addition, future studies including both sexes, different dose levels, and a more comprehensive physicochemical characterization of the microencapsulated extract within the same experimental design would further expand the interpretation and translational relevance of these findings. Future studies should also assess the bioavailability of the microencapsulated extract and further investigate the mechanistic pathways underlying the observed effects.

When comparing the different jabuticaba formulations evaluated, all the interventions demonstrated beneficial effects against metabolic alterations induced by an HFHF diet. Although the overall metabolic improvements were comparable among the formulations, some responses differed according to the food matrix. Overall, the consumption of the whole fruit and the peel resulted in largely similar metabolic and oxidative outcomes, indicating that both food matrices are effective in modulating systemic disturbances associated with HFHF feeding. The whole fruit was associated with greater antioxidant enzyme activity, whereas the microencapsulated extract produced a more pronounced reduction in adipocyte area and was the only formulation associated with increased brown adipose tissue mass. These effects likely reflect the combined action of phenolic compounds, dietary fiber, and other bioactive constituents naturally present in the fruit matrix, as well as differences introduced by processing and formulation.

Notably, although reductions in adipocyte area were observed across all the jabuticaba treatments, the microencapsulated peel extract induced a more pronounced decrease, in parallel with an increase in brown adipose tissue mass. In contrast, the whole fruit exhibited greater antioxidant enzyme activity, highlighting that the different formulations showed distinct strengths depending on the biological parameter evaluated. Because phenolic bioavailability and stability were not directly measured, these formulation-dependent differences cannot be attributed to improved delivery of bioactive compounds. Therefore, rather than indicating an overall superiority of the microencapsulated formulation, the present findings suggest that whole fruit, peel, and microencapsulated peel extract each provide beneficial, yet partially distinct, metabolic and redox-related effects. Microencapsulation represents a promising technological strategy for the delivery of jabuticaba-derived phenolic compounds. However, additional studies evaluating phenolic bioavailability, molecular mechanisms, and long-term efficacy are necessary to better understand the specific biological effects associated with this formulation and to define its potential applications in functional foods and nutraceuticals.

## 4. Materials and Methods

### 4.1. Jabuticaba, Peel, and Microencapsulated Peel Extract

*Plinia* sp. fruits were purchased directly from local rural producers in the city of Jequeri (20°31′32.2″ S 42°39′11.2″ W), Minas Gerais, Brazil, during the harvest season. The fruits were cultivated under organic farming practices with no use of agrochemicals, as confirmed by the producers, and were therefore considered organically produced for the purposes of this study. All the fruits were harvested during a single collection period to avoid batch-to-batch variability. To ensure representative sampling, mature fruits were collected from different trees within the same cultivation area and subsequently pooled before processing. Only fully ripe fruits were selected based on uniform dark-purple coloration, and fruits showing mechanical damage, fungal contamination, or visible peel deterioration were excluded. After collection, the fruits were carefully inspected, sanitized, and homogenized in order to minimize biological variability while preventing inter-batch variation. Finally, the fruits were then manually separated into: (1) jabuticaba whole fruit and (2) jabuticaba peel, and both were submitted to freeze-drying.

To develop the extract, the freeze-dried jabuticaba peels were ground in a knife mill (Brabender^®^, Duisburg, Germany) with a 1.0 mm stainless steel sieve until a homogeneous powder was obtained. An initial compositional screening was conducted to compare the phenolic content of the whole fruit and the peel. This preliminary analysis demonstrated that the peel contained higher concentrations of total phenolic compounds and anthocyanins than the whole fruit. Based on these findings, the phenolic extract was produced exclusively from the peel fraction in order to concentrate the bioactive compounds of interest. The jabuticaba peel powders were extracted as previously described with modifications [47]. Thus, the jabuticaba peel powders were extracted using a hydroethanolic solution (ethanol:water, 50:50 *v*/*v*), at a solid-to-solvent ratio corresponding to 15% (*w*/*v*) of jabuticaba peel powder. The extraction was carried out in Falcon tubes for 2 h under continuous agitation in a metabolic bath at room temperature, with the samples protected from light to prevent degradation of light-sensitive compounds. The extract was then centrifuged at 3500 rpm for 10 min, and the supernatant was vacuum-filtered through a 0.22 μm membrane filter. The filtrate was then concentrated by evaporation under reduced pressure at 40 °C for 20 min in a rotary evaporator coupled to a vacuum pump.

For microencapsulation, maltodextrin was used as the wall material at a concentration of 30%, previously diluted in distilled water and homogenized on a magnetic stirrer for 10 min. Subsequently, the extract was submitted to spray drying (SD), model MSD (LabMaq, Ribeirão Preto, SP, Brazil), equipped with a chamber and cyclone, with a 1 mm diameter pressure nozzle [48]. The drying parameters used were: inlet air temperature 170 ± 1 °C; outlet air temperature 88.5 ± 1.45 °C; blower air flow rate, 2.2 m^3^/min^−1^ to 2.8 m^3^/min^−1^; and product inlet flow rate, 0.73 kg h^−1^. The phenolic microencapsulated peel extract used in the present study corresponded to the same production batch previously characterized by our research group in terms of its physicochemical, technological, and phytochemical properties [16]. The microencapsulated extract, as well as the freeze-dried peel and freeze-dried whole fruit powders obtained, were stored in hermetically sealed laminated polyethylene containers until their incorporation into the experimental diets.

### 4.2. Ultra-High-Performance Liquid Chromatography with Photodiode Array (UPLC-PDA) Analysis

The analyses were carried out using a UPLC-PDA equipped with a quaternary solvent manager, an autosampler manager and a column manager (Waters, Acquity H-Class, Milford, MA, USA). Chromatographic separation was performed on a Kinetex^®^ C18 column (2.6 μm, 100 mm × 4.6 mm—Phenomenex, Torrance, CA, USA) using a binary mobile phase composed of water (A) and acetonitrile (B), both acidified with formic acid (1%) in the following gradient mode: B (%): 0–1 min (3–13%), 1–2 min (13–15%), 2–2.5 min (15%), 2.5–3 min (15–18%), 3–3.5 min (18–20%), 3.5–4 min (20–25%), 4–4.5 min (25–27%), 4.5–5 min (27–28%), 5–6 min (28–30%), 6–7 min (30%), 7–9 min (30–40%), 9–10 min (40–60)%, 10–12 min (60–3%). The injection volume was 10 μL, the column temperature was maintained at 47 °C, and the flow rate was 0.7 mL min^−1^.

Samples were dissolved in methanol/water (50%/50%) using an ultrasonic bath (P60H, Elmasonic, Singen, Germany) for 7 min in sweep mode at 37 kHz and 100 W. Compounds were identified based on their UV-Vis absorption spectra in the range of 200–500 nm and by comparing retention times with those of standard solutions. Minor differences in retention times among chromatograms may be observed because samples were analyzed in independent HPLC runs. Compound identification was confirmed by comparison with authentic standards and UV–Vis spectral characteristics. Quantification of cyanidin-3-glucoside and ellagic acid was performed using external calibration curves prepared from authentic reference standards. Empower 3 software (Waters, Alliance, Milford, MA, USA) was used for data processing.

### 4.3. Animals and Ethics Statement

Forty-five 8-week-old male C57BL/6 mice (15–17 g) were provided by the Central Animal Facility of the Universidade Federal de Viçosa (UFV), Minas Gerais, Brazil. The animals were housed collectively (3 animals per cage) in polypropylene cages covered with metal grates (330 × 200 × 130 mm) in a temperature-controlled environment (21 ± 3 °C) with an automatically controlled 12 h photoperiod. They underwent a one-week acclimation period, with access to distilled water and ad libitum commercial diet. The study was approved by the UFV Animal Use Ethics Committee (protocol 10/2023) and conducted in strict accordance with the ethical guidelines of the Guide for the Care and Use of Laboratory Animals (Guide for the Care and Use of Laboratory Animals, 2011).

### 4.4. Experimental Design and Diet Formulation

The experiment was divided into two phases. In Phase I, the induction of metabolic changes, the animals were randomly divided into two groups: a standard control group (*n* = 9), which received the AIN-93M diet [49], and an HFHF group (*n* = 36), which received a diet rich in saturated fatty acids and fructose (60%) [50]. This phase lasted for 6 weeks. At the end of the 6-week induction period, the measurements (abdominal circumference and nasoanal length) were collected to calculate the Lee index.

The control group fed the AIN-93 diet was included as a physiological reference, providing baseline parameters for comparison with the groups fed the HFHF diet and allowing for the interpretation of metabolic changes induced by diet and treatment. Based on the observed metabolic alterations, the second phase of the study was conducted exclusively with animals previously exposed to the HFHF diet, as the goal was to evaluate the effects of jabuticaba in the context of pre-existing metabolic changes. Phase II, also lasting 6 weeks, began after the induction phase. The animals in the HFHF group were randomly redistributed into four groups (*n* = 9 animals/group): G1) HFHF diet (HFHF); G2) HFHF diet + freeze-dried whole jabuticaba fruit (HFJ); G3) HFHF diet + freeze-dried jabuticaba peel (HFP); and G4) HFHF diet + microencapsulated peel extract (HFE).

Experimental diets were designed according to Reeves, Nielsen, and Fahey [16] for the AIN-93M diet (standard control group) and Martinez et al. [49] for the HFHF diet (obese group). The HFHF group received a diet with increased lipid content, consisting of 4% soybean oil and 31% lard, and 20% fructose. The concentration of total phenolics in the jabuticaba treatments was determined using the Folin–Ciocalteu colorimetric method. Thus, jabuticaba treatments were added to the diet in adequate quantities to provide 200 mg of polyphenols per 100 g of diet (Table 2). Therefore, no variation in polyphenol content occurred among the test diets.

The selected dosage was based on previously published studies investigating jabuticaba or its polyphenol-rich fractions in models of diet-induced metabolic dysfunction, which have employed comparable concentrations of phenolic compounds [12,15,51,52]. In addition, diet formulations were defined after detailed product characterization and proximate composition analyses of the powdered jabuticaba fractions. Based on these data, ingredient substitutions were carefully performed to guarantee equivalent macronutrient distribution among groups. Thus, the contributions of carbohydrates, proteins, lipids, and fiber from each jabuticaba matrix were quantified, and macronutrients from other dietary sources were proportional. The experimental diets were protected from light using stainless steel lids, and the treatment-containing pellets were replaced every other day to preserve polyphenol stability and minimize oxidative degradation.

All the ingredients were weighed on a semi-analytical balance (BK 2000, Gehaka, São Paulo, Brazil), mixed manually, sieved, and homogenized in an industrial mixer (Leme) for fifteen minutes. After preparation, the diets were packaged in dark polyethylene bags, properly labeled, and stored in a freezer (−18 ± 1 °C) to minimize fatty acid oxidation.

### 4.5. Body Composition and Biochemical Analysis

Murine biometric measurements and food consumption of the animals were recorded weekly. Nasoanal length (NAL) and abdominal perimeter were measured using a flexible measuring tape placed around the abdominal region of the animals at a standardized anatomical location. The Lee index was calculated by the ratio between the cubic root of body weight and the nasal-anal length, using the following formula: $\sqrt[{3}]{\frac{weight\;(g)}{NAL\;(cm)}}$ [53].

Animal euthanasia was performed at the end of phase II in an appropriate environment by a trained team. After a 6 h fast, the animals were anesthetized with 3% inhaled isoflurane until deep sedation was achieved. Physiological parameters were then assessed to confirm adequate anesthesia before total exsanguination via cardiac puncture. Adipose tissue depots collected included inguinal subcutaneous adipose tissue, brown, epididymal, and visceral fat. Epididymal white adipose tissue (eWAT) compartments were removed, weighed, and rapidly fragmented into four parts. Three fragments were frozen in liquid nitrogen and stored at −80 °C for later analysis, and the remaining fragment was fixed and used for histological analysis.

For the determination of adiposity index was calculated as the sum of fat pad weights (inguinal, brown, epididymal, and visceral) divided by the final body weight and expressed as a percentage [54]. Glucose, total cholesterol, high-density lipoprotein cholesterol (HDL), low-density lipoprotein cholesterol (LDL), and triglycerides (TG) levels were measured in serum by colorimetric methods using commercially available kits (Bioclin^®^, Belo Horizonte, Brazil) according to the manufacturer’s instructions.

### 4.6. Assessment of Glucose Tolerance

The intraperitoneal glucose tolerance test (iGTT) was conducted in the last week (Phase II) of the experiment, after a 6 h fast. Blood glucose levels were measured with a portable glucometer (Accu-Chek^®^ Roche, Mannheim, Germany) using appropriate test strips. For the iGTT, a 50% D-glucose solution (2 g·kg^−1^ body weight) was injected into the peritoneal cavity after a 6 h fast. Blood glucose levels were measured at baseline (time 0) and after 30, 60, 90, and 120 min from the tail vein. The area under the glucose curve was calculated using GraphPad Prism 9 (GraphPad Software, San Diego, CA, USA).

### 4.7. Endogenous Antioxidant Defense and Oxidative Stress Markers

For this analysis, 100 mg of adipose tissue was homogenized in phosphate-buffered saline (PBS) and centrifuged at 3500× *g* for 10 min at 4 °C. The resulting supernatant was used for the determination of enzymatic antioxidant activities and oxidative stress markers. Total protein concentration was measured using the Bradford assay [55], and all enzymatic activities were normalized to protein content.

#### 4.7.1. Superoxide Dismutase (SOD)

The SOD activity was determined based on its ability to neutralize superoxide radicals, thereby inhibiting pyrogallol auto-oxidation, as described by Madesh and Balasubramanian [56]. Briefly, samples, standards, and blanks were incubated at 37 °C for 5 min, and absorbance was measured at 320 nm using a spectrophotometer (ThermoScientific^®^ Multiskan GO, Thermo Fisher Scientific Oy, Vantaa, Finland).

#### 4.7.2. Catalase (CAT)

The CAT activity was evaluated by measuring the decomposition of hydrogen peroxide into water and oxygen, following the protocol described by Hadwan & Abed [57]. Supernatants were diluted 1:10 in distilled water, mixed with phosphate buffer (pH 7.0) and H_2_O_2_ (40 µL of 30% H_2_O_2_ per 25 mL), and absorbance was recorded at 374 nm.

#### 4.7.3. Glutathione-S-Transferase (GST)

The GST activity was assessed by monitoring the conjugation of reduced glutathione (GSH) to 1-chloro-2,4-dinitrobenzene (CDNB), according to Habig and Jakoby [58]. Following centrifugation of the homogenate, CDNB and GSH were added to the supernatant, and absorbance was measured at 340 nm using a spectrophotometer (ThermoScientific^®^ Multiskan GO).

#### 4.7.4. Malondialdehyde (MDA)

Lipid peroxidation was estimated by measuring MDA as thiobarbituric acid-reactive substances (TBARS), following Buege and Aust [59]. Supernatants were reacted with TBA under acidic heating, forming a pink chromogen. Absorbance was recorded at 535 nm, and MDA concentrations were calculated using a standard curve prepared with 1,1,3,3-tetramethoxypropane (TMPO).

#### 4.7.5. Protein Carbonyls (PCNs)

Protein carbonyl content was determined using the 2,4-dinitrophenylhydrazine (DNPH) assay, based on the formation of hydrazone derivatives from carbonyl groups, as described by Levine et al. [60]. Absorbance was measured at 370 nm.

#### 4.7.6. Nitric Oxide (NO)

The NO production was indirectly estimated by measuring nitrite/nitrate levels using the Griess reaction [61]. Supernatants (50 µL) were incubated with 100 µL of Griess reagent (1% sulfanilamide, 0.1% N-(1-naphthyl)ethylenediamine, 2.5% H_3_PO_4_) for 10 min at room temperature. Absorbance was read at 570 nm, and NO concentrations were calculated using a sodium nitrite standard curve (0–125 µM).

### 4.8. Histomorphometry Analyses

Samples of adipose tissue extracted from mice were cut into 2 cm^3^ pieces and trimmed. The fixation process was performed by maintaining the adipose tissue samples in a 10% buffered formalin solution for 24 h. The fixed adipose tissue fragments were dehydrated in an increasing series of ethanol (70, 80, 90%, and absolute), cleared in xylene, and embedded in paraffin. 5 μm sections were obtained in semi-series, using one section every 10 μm to avoid repeated analyses of the same histological area. The sections were stained with hematoxylin and eosin (H&E) for morphometric analyses. After the staining procedures, the slides were mounted with Entellan^®^ (Merck, Germany). Images were captured with a 10X objective. Histological fields and image acquisition were performed using a photomicroscope (Olympus BX53, Tokyo, Japan) connected to a digital camera (Olympus DP73, Tokyo, Japan). The images of the histological sections were analyzed using Adiposoft v1.16, a software for the analysis of white adipose tissue cellularity in histological sections, an open-source ImageJ plugin (ImageJ^®^ V 1.8.0 software system). For counting and measuring the area of adipocytes, 10 fields per animal were captured. The average of these values was calculated and used in the statistical analyses. All the histological analyses were performed in a blinded manner by an evaluator who was unaware of the experimental group assignments for the animals.

### 4.9. Statistical Analysis

Samples were assessed for normality using the Shapiro–Wilk test. Statistically significant differences between groups were determined using analysis of variance (ANOVA). Post hoc comparisons were performed using the Newman–Keuls test to allow multiple pairwise comparisons among the experimental groups. *p*-values less than 0.05 (*p* < 0.05) were considered statistically significant. Data were expressed as mean ± standard deviation (SD). The correlation between oxidative stress biomarkers, biochemical variables, histological parameters, and adipose tissue weight was analyzed by Pearson’s correlation test. The variables that did not show a normal distribution were transformed using the logarithmic scale. The statistical analyses and graphs were performed using GraphPad Prism^®^ statistical software, version 9.0 (GraphPad Software Inc., San Diego, CA, USA).

## 5. Conclusions

Jabuticaba, regardless of its formulation, is associated with improvements in parameters related to adipose tissue alterations induced by a diet rich in fat and fructose, reducing visceral fat accumulation, improving metabolic and biochemical profiles, attenuating oxidative stress, and limiting adipocyte hypertrophy. These findings should be interpreted as referring to *Plinia* sp., as the plant material was not formally botanically authenticated. The microencapsulated peel extract showed comparable or superior effects in specific parameters, suggesting that microencapsulation represents a promising strategy to expand the functional application of phenolic extracts. These findings support the relevance of this technological approach while indicating that further studies should focus on elucidating the mechanisms involved and confirming its efficacy in different experimental and clinical settings.

## Figures and Tables

**Table 1:** Effect of jabuticaba consumption, its peel, and microencapsulated peel extract on murinometric and consumption indicators, and biochemical variables in the experimental groups.

Variables	HFHF	HFJ	HFP	HFE
Murinometric and food consumption indicators				
Initial weight (g)	14.27 ± 1.48 ^a^	15.11 ± 1.22 ^a^	14.47 ± 1.53 ^a^	15.49 ± 1.12 ^a^
Final weight (g)	27.03 ± 1.01 ^a^	24.63 ± 1.00 ^b^	24.43 ± 0.77 ^b^	24.05 ± 1.37 ^b^
Food intake mean (g/day)	2.01 ± 0.16 ^a^	2.12 ± 0.21 ^a^	2.09 ± 0.17 ^a^	2.12 ± 0.26 ^a^
Energy intake mean (kcal/day)	10.41 ± 0.78 ^a^	10.99 ± 1.10 ^a^	10.87 ± 0.96 ^a^	10.99 ± 1.38 ^a^
Lee index	336.7 ± 9.83 ^a^	324.00 ± 3.72 ^b^	321.70 ± 4.45 ^b^	322.60 ± 7.47 ^b^
Biochemical variables in serum				
Blood glucose (mg/dL)	262.20 ± 14.79 ^a^	232.90 ± 16.67 ^b^	233.00 ± 8.86 ^b^	232.50 ± 12.22 ^b^
Total cholesterol (mg/dL)	95.33 ± 8.26 ^a^	91.47 ± 4.51 ^a^	93.19 ± 2.86 ^a^	91.06 ± 3.67 ^a^
LDL-C (mg/dL)	60.11 ± 2.48 ^a^	48.10 ± 1.18 ^b^	46.66 ± 6.74 ^b^	46.65 ± 6.22 ^b^
HDL-C (mg/dL)	48.25 ± 6.42 ^b^	57.02 ± 2.17 ^a^	55.51 ± 5.30 ^a^	58.11 ± 4.54 ^a^
Triglycerides (mg/dL)	82.77 ± 2.71 ^a^	67.74 ± 1.38 ^c^	72.60 ± 3.82 ^b^	70.07 ± 2.74 ^bc^

Values are expressed as means ± standard deviation (SD). *n* = 9/group. a–c Treatment group means not indicated by the same letter are significantly different (*p* < 0.05) according to the Newman–Keuls test. HFHF: diet rich in saturated fatty acids and fructose; HFJ: diet rich in saturated fatty acids and fructose + freeze-dried whole jabuticaba fruit; HFP: diet rich in saturated fatty acids and fructose + freeze-dried jabuticaba peel; HFE: diet rich in saturated fatty acids and fructose + microencapsulated peel extract. LDL-C: low-density lipoprotein; HDL-C: high-density lipoprotein.

**Table 2:** Composition of experimental diets.

Ingredients (g·kg^−1^)	AIN-93M	HFHF	HFJ	HFP	HFE
Freeze-dried jabuticaba	0.00	0.00	64.00	0.00	0.00
Freeze-dried peel	0.00	0.00	0.00	34.00	0.00
Microencapsulated extract	0.00	0.00	0.00	0.00	203.00
Albumin *	150.00	150.00	146.75	148.33	150.00
Sucrose	100.00	28.64	28.64	28.64	0.00
Soybean oil	40.00	40.00	36.13	37.82	39.39
Lard	-	310.00	310.00	310.00	310.00
Fructose	-	200.00	200.00	200.00	198.31
Cellulose	50.00	50.00	43.18	42.75	50.00
Mineral mix	35.00	35.00	35.00	35.00	35.00
Vitamin mix	10.00	10.00	10.00	10.00	10.00
L-cystine	1.80	1.80	1.80	1.80	1.80
Choline bitartrate	2.50	2.50	2.50	2.50	2.50
Macronutrients					
Protein (%)	13.00	9.30	9.30	9.30	9.40
Carbohydrate (%)	77.00	30.60	30.30	30.80	30.00
Lipid (%)	10.00	60.10	60.40	59.90	60.60
Caloric density (Kcal/g)	3.69	5.24	5.22	5.26	5.20

* Albumin based on 81.02% protein content. AIN-93M: standard rodent diet; HFHF: diet high in saturated fat and fructose; HFJ: diet high in saturated fat and fructose + freeze-dried whole jabuticaba fruit; HFP: diet high in saturated fat and fructose + jabuticaba peel; HFE: diet high in saturated fat and fructose + microencapsulated peel extract.

## Data Availability

The data will be shared upon reasonable request to the corresponding author due to ethical restrictions related to the use of animals in research.

## Abbreviations

The following abbreviations are used in this manuscript:

AIN-93M	Standard diet for rodents
BSA	Body surface area
CAT	Catalase
DNPH	2,4-dinitrophenylhydrazine
eWAT	Epididymal white adipose tissue
GSH	Reduced glutathione
GST	Glutathione S-transferase
HDL	High-density lipoprotein cholesterol
HFE	Diet rich in saturated fatty acids and fructose + microencapsulated jabuticaba peel extract
HFHF	High-fat high-fructose
HFJ	Diet rich in saturated fatty acids and fructose + freeze-dried whole jabuticaba fruit
HFP	Diet rich in saturated fatty acids and fructose + freeze-dried jabuticaba peel
iGTT	Intraperitoneal glucose tolerance test
LDL	Low-density lipoprotein cholesterol
MDA	Malondialdehyde
NAL	Nasoanal length
NADPH	Nicotinamide adenine dinucleotide phosphate (reduced form)
NO	Nitric oxide
PBS	Phosphate-buffered saline
PCN	Protein carbonyls
ROS	Reactive oxygen species
SD	Spray drying
SOD	Superoxide dismutase
TBARS	Thiobarbituric acid reactive substances
TG	Triglycerides
TMPO	1,1,3,3-tetramethoxypropane
UPLC-PDA	Ultra-high-performance liquid chromatography with photodiode array
vWAT	Visceral white adipose tissue
